# Selective killing of cancer cells by nanoparticle-assisted ultrasound

**DOI:** 10.1186/s12951-016-0194-9

**Published:** 2016-06-14

**Authors:** Olga K. Kosheleva, Tsung-Ching Lai, Nelson G. Chen, Michael Hsiao, Chung-Hsuan Chen

**Affiliations:** Genomics Research Center, Academia Sinica, Taipei, 211 Taiwan; Department of Electrical and Computer Engineering, Institute of Biomedical Engineering, College of Electrical and Computer Engineering, National Chiao-Tung University, Hsinchu, 300 Taiwan

**Keywords:** Cancer cells, Nanoparticles, Ultrasound therapy

## Abstract

**Background:**

Intense ultrasound, such as that used for tumor ablation, does not differentiate between cancerous and normal cells. A method combining ultrasound and biocompatible gold or magnetic nanoparticles (NPs) was developed under in vitro conditions using human breast and lung epithelial cells, which causes ultrasound to preferentially destroy cancerous cells.

**Results:**

Co-cultures of BEAS-2B normal lung cells and A549 cancerous lung cells labeled with green and red fluorescent proteins, respectively, were treated with focused ultrasound beams with the addition of gold and magnetic nanoparticles. There were significantly more necrotic A549 cells than BEAS-2 cells when gold nanoparticles were added to the culture medium [(50.6 ± 15.1) vs. (7.4 ± 2.9) %, respectively, P < 0.01]. This selective damage to cancer cells was also observed for MDA-MB231 breast cancer cells relative to MCF-10A normal breast cells after treatment with magnetic nanoparticles.

**Conclusions:**

The data obtained for different cell lines indicate that nanoparticle-assisted ultrasound therapy (NAUT) could be an effective new tool for cancer-specific treatment and could potentially be combined with conventional methods of cancer diagnosis and therapy to further increase the overall cancer cure rate.

## Background

Cancer is a leading cause of death worldwide. Many cancer patients die as a result of the severe side effects of chemotherapy or from a drug-resistance-related relapse after the original treatment. It has been established that a typical tumor has a high degree of heterogeneity and can contain more than 100 cell types. If a targeted drug is used, certain types of cancer cells may survive and become dominant in the tumor, eventually making the drug ineffective.

If adequate physical treatment is applied, it should reduce not only the side effects but also the growth of drug-resistant cancer cells. Radiation therapy is one of the standard physical methods of cancer treatment. There are various types of radiation therapies, including electromagnetic ionizing radiation (gamma-rays and X-rays), elementary particle ionizing beam radiation (electrons, protons and neutrons), and non-ionizing radiation (photons, microwaves and radio waves). The ionizing radiation is intended to be directed only at the tumor. However, radiation is difficult to focus; therefore, it also affects normal tissues when it passes through a patient’s body. Normal cells are affected by ionizing radiation, which produces undesirable side effects [1]. Moreover, ionizing radiation itself may cause DNA mutation in normal cells, causing these cells to become cancerous. Light is used as non-ionizing radiation in photodynamic therapy for tumors, and this method relies on the ability of certain photosensitizing dyes to absorb light and convert the energy into cytotoxic molecules, including singlet oxygen [2]. The main limitations of photodynamic methods are solid tumor hypoxia [3] and relatively low penetration. The other disadvantage of the method is the need to avoid light exposure for an extensive period after the treatment. Other non-ionizing radiation therapies are mainly based on hyperthermia in tumors as a result of the higher sensitivity of tumor cells to heat than their normal counterparts [4]. Hyperthermia has been induced by gold nanoparticle-assisted laser ablation [5], magnetic nanoparticle rotation under an alternating magnetic field [6], and high-intensity focused ultrasound (HIFU) [7].

Unlike other methods, focused ultrasound is the only non-invasive technology that can be used for local tumor ablation deep inside the body without causing severe harm to overlying skin or adjacent connective tissues. For conventional HIFU transducers, the absorption efficiencies of the acoustic energy are generally similar for normal and malignant tissues such that precise focusing of ultrasound beams is required for selective tumor ablation. Moreover, normal tissue near the tumor region can still be damaged by high-intensity ultrasound waves reflected from interfaces [7]. Therefore, a new physical method that does not cause DNA mutations or destroy normal cells is highly desirable. To overcome the drawbacks of HIFU, we developed a new technique based on an HIFU instrument and biocompatible gold and magnetic nanoparticles (NPs) in this study. Our results showed that adding NPs to culture media allowed for the use of lower ultrasound (US) intensities to treat and kill cells compared with those used in conventional US treatment. Nanoparticle-assisted ultrasound therapy (NAUT) allowed for the selective killing of malignant breast and lung epithelial cells over their normal counterparts.

## Results and discussion

### Ultrasound treatment of lung cells

#### Ultrasound treatment of separate cell monolayers

**Fig. 1:**
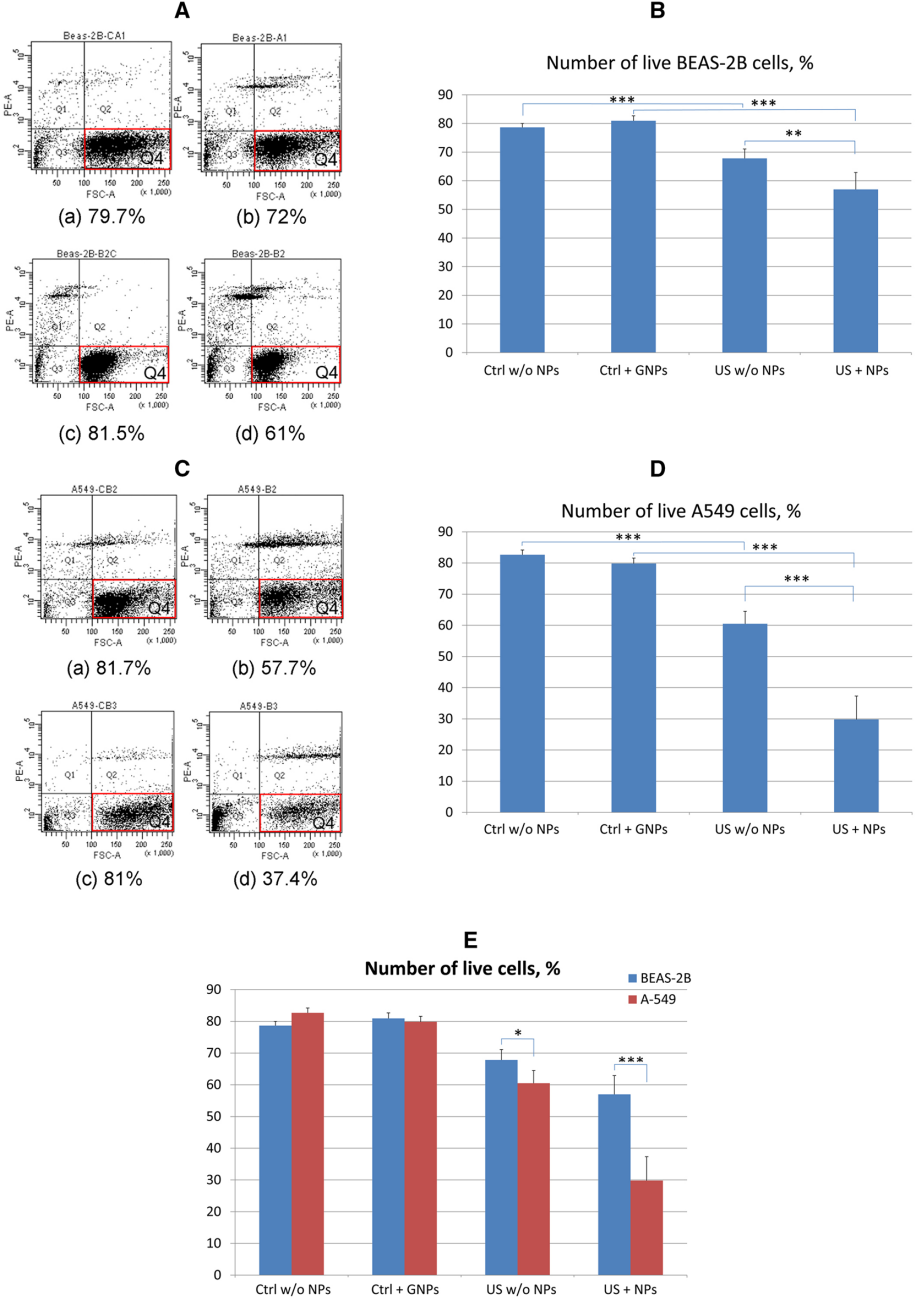
Flow cytometry data for BEAS-2B normal lung cells (**A**) and A549 lung cancer cells (**C**) after US treatment with gold NPs and the corresponding numbers of live BEAS-2B (**B**, **E**) and A549 (**D**, **E**) cells. **A**, **C** Control (untreated) cells without NPs (*a*); control (untreated) cells with gold NPs (*c*); cells treated with US without NPs (*b*); cells exposed to US with gold NPs (*d*). *represents *P* < 0.05, **represents *P* < 0.01, ***represents *P* < 0.001

The effect of ultrasound (US) and gold NPs on cancerous (A549) and normal (BEAS-2B) lung cells, as analyzed by counting viable cells (Q_4_) using flow cytometry, is shown in Fig. 1.

Flow cytometry data for the propidium iodide viability test on BEAS-2B and A549 cells are provided in Fig. 1A, C, respectively. The mean numbers of viable BEAS-2B and A549 cells for five similar experiments, with calculated P values, are shown in Fig. 1B–E and Table 1.

**Table 1:** Percentage of live BEAS-2B and A549 cells in separate monolayers treated with US and gold NPs averaged for five similar experiments

Cell line	Percentage of live cells not treated with NPs	Percentage of live cells treated with NPs	Percentage of live cells treated with US alone	Percentage of live cells treated with US and NPs
BEAS-2B	78.6 ± 1.4	80.9 ± 1.8	67.8 ± 3.3	57 ± 6.0
A549	82.7 ± 1.5	79.8 ± 1.8	60.5 ± 3.9	29.8 ± 7.5

The results demonstrate a slight decrease in the percentage of live cells from (67.8 ± 3.3) to (57 ± 6.0) % when gold NPs were added to US-treated normal BEAS-2B cells (Fig. 1B, E; Table 1). Interestingly, the percentage of live A549 cancer cells decreased from (60.5 ± 4.0) % for US-treated cells to (29.8 ± 7.5) % for the combination of US with NPs (Fig. 1D, E; Table 1). These results indicate that A549 lung cancer cells are more sensitive to the damage caused by US than BEAS-2B normal lung cells are. The addition of gold NPs to cell cultures leads to a significant increase in cancer cell death and shows the synergistic damaging effect of US and nanoparticles. The BEAS-2B normal cells appear to be more resistant to this effect.

#### Ultrasound treatment of cell co-cultures

**Fig. 2:**
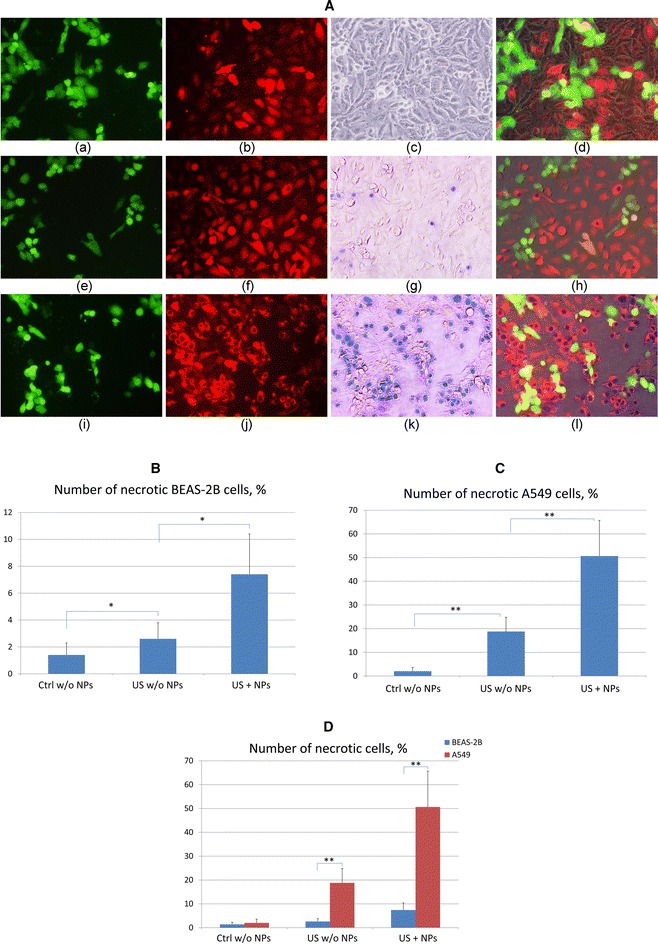
Phase-contrast and fluorescence images of trypan blue stained BEAS-2B and A549 co-cultures with and without US exposure (**A**) and the corresponding numbers of necrotic BEAS-2B (**B**, **D**) and A549 (**C**, **D**) cells. **A** Control (untreated) cells (*a*–*d*); cells treated only with US (*e*–*h*); cells treated with US and gold NPs (*i*–*l*); fluorescence images of normal cells (*green*) (*a*, *e*, *i*) and cancer cells (*red*) (*b*, *f*, *j*); phase-contrast images of the co-culture (*c*, *g*, *k*); merged fluorescence and phase-contrast images (*d*, *h*, *l*). *represents *P* < 0.05, and **represents *P* < 0.01

To compare the effect of ultrasound irradiation on normal and cancerous cells under identical experimental conditions, BEAS-2B and A549 human lung cells were co-cultured and modified with fluorescent green and red proteins, respectively. Monolayers of the co-culture were then treated with US with and without the addition of gold NPs. This experiment sought to eliminate possible differences due to the variations in US treatments and culture conditions for cancerous and normal cells. To estimate the cell damage, we analyzed trypan blue-stained cells under an optical microscope. Fluorescent and phase-contrast images of trypan blue-stained BEAS-2B/A549 co-culture treated with different combinations of US and gold NPs are shown in Fig. 2A. The mean percentage of necrotic BEAS-2B and A549 cells for five similar experiments is presented in Fig. 2B, D and in Table 2.

**Table 2:** Percentage of necrotic BEAS-2B and A549 cells in co-culture treated with a combination of US and gold NPs

Cell line	No NP and no US	US but no NP	US and NPs
BEAS-2B	1.4 ± 0.9	2.6 ± 1.2	7.4 ± 2.9
A549	2 ± 1.6	18.8 ± 5.9	50.6 ± 15.1

The number is the average for five similar experiments

Phase-contrast and fluorescence images of the control (untreated) samples without gold NPs are shown in Fig. 2Aa–d. Cells with dark- or blue-colored nuclei are necrotic. Only a few dead cells were observed in the control samples of either cell line. A few dead BEAS-2B cells are shown in Fig. 2Ae–h; these cells underwent US treatment without the addition of nanoparticles (Fig. 2B, D; Table 2). Under these conditions, the number of necrotic A549 cells was approximately (18.8 ± 6.0) % (Fig. 2C, D; Table 2). Fluorescence images of the co-culture treated with the combination of ultrasound irradiation in the presence of gold NPs are shown in Fig. 2Ai–l. The combined treatment of cells resulted in (7.4 ± 3.0) and (50.6 ± 15.1) % necrotic BEAS-2B and A549 cells, respectively (Fig. 2B–D; Table 2). Thus, the addition of gold NPs to the culture medium immediately before the US exposure enhances the damage to both types of lung cells. However, the synergistic cytotoxic effects of US and NPs are more pronounced on A-549 lung cancer cells under co-culture conditions than on their normal counterparts (BEAS-2B cells).

### Ultrasound treatment of breast cells

**Fig. 3:**
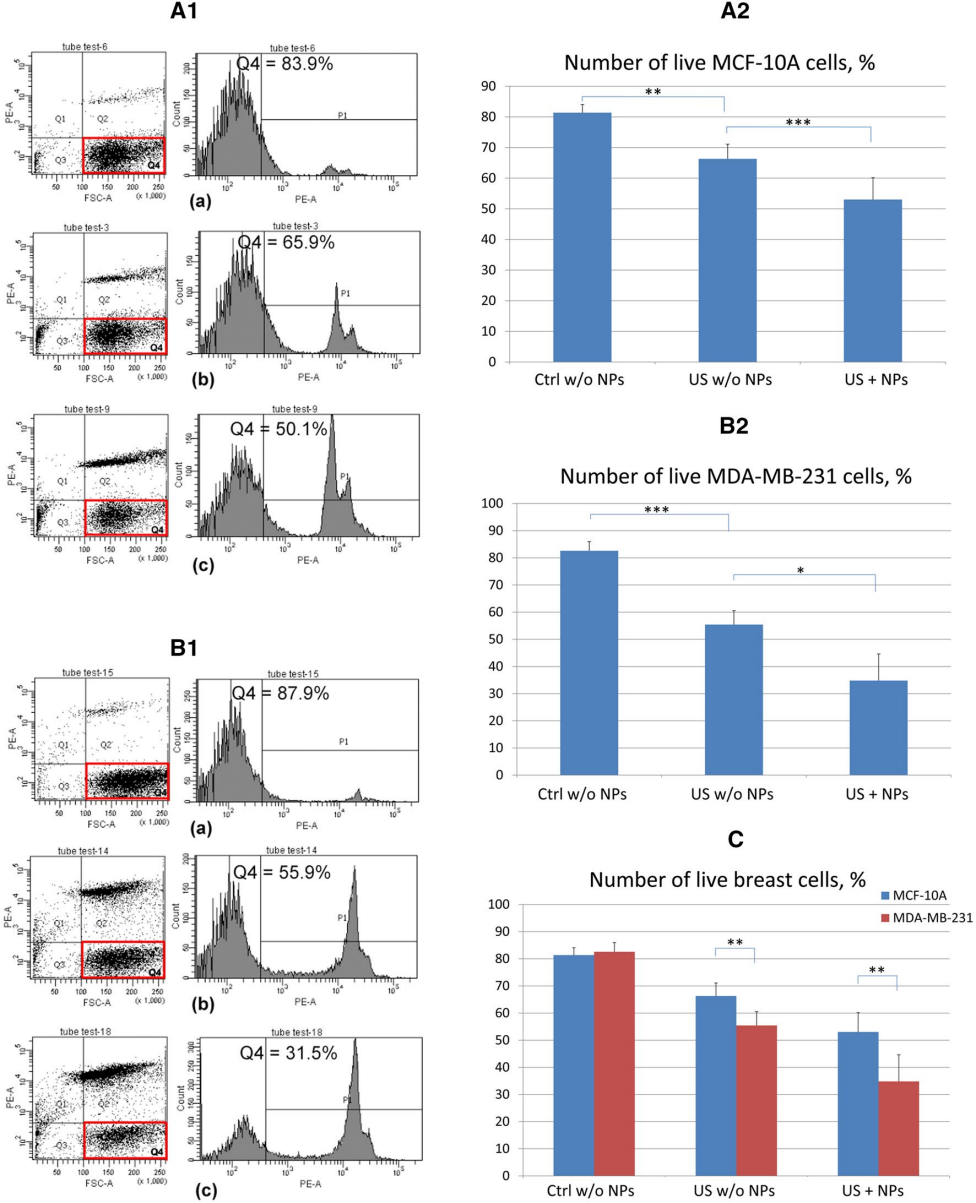
Flow cytometry data for MCF-10A normal breast cells (**A**) and MDA-MB-231 breast cancer cells (**C**) with/without US treatment and magnetic NPs and the corresponding numbers of live MCF-10A (**B**, **E**) and MDA-MB-231 (**D**, **E**) cells. **A**, **C** Control (untreated) cells not treated with NPs (*a*); cells treated with US alone (*b*); cells exposed to US and magnetic NPs (*c*). *represents *P* < 0.05, **represents *P* < 0.01, ***represents *P* < 0.001

A similar selective killing effect on cancer cells was observed when a monolayer of MCF-10A normal breast cells and MDA-MB-231 breast cancer cells were treated with a combination of US and biocompatible magnetic nanoparticles. The flow cytometry data and the mean number of live cells for five similar experiments are shown in Fig. 3 and Table 3.

**Table 3:** Percentage of live MCF-10A and MDA-MB-231 cells in separate monolayers treated with US and magnetic NPs, averaged for five similar experiments

Cell line	Percentage of live control cells not treated with NPs	Percentage of live cells treated with US alone	Percentage of live cells treated with US and NPs
MCF-10A	81.4 ± 2.7	66.3 ± 4.8	53 ± 7.1
MDA-MB-231	82.6 ± 3.4	55.4 ± 5.1	34.8 ± 9.8

The flow cytometry data for MCF-10A normal cells and MDA-MB-231 malignant cells are provided in Fig. 3A, C, respectively. The number of live cells in Q_4_ decreased after US exposure from (81.4 ± 2.7) % for control samples to (66.3 ± 4.8) % for MCF-10A cells (Fig. 3B, E; Table 3) and from (82.6 ± 3.4) % for control samples to (55.4 ± 5.1) % for MDA-MB-231 cells (Fig. 3D, E; Table 3). Compared with cells treated with US only, the addition of magnetic NPs to the culture media immediately before US exposure resulted in a reduction in the percentage of live MCF-10A cells, from (66.3 ± 4.8) to (53 ± 7.1) % (Fig. 3B, E; Table 3), and a decrease in the number of live MDA-MB-231 cells, from (55.4 ± 5.1) to (34.8 ± 9.8) %. These findings demonstrate that lung and breast cells exhibit similar sensitivity to US irradiation, as well as to combined treatment with US and gold or magnetic NPs.

### Transmission electron microscopy images of ultrasound treated breast cells

**Fig. 4:**
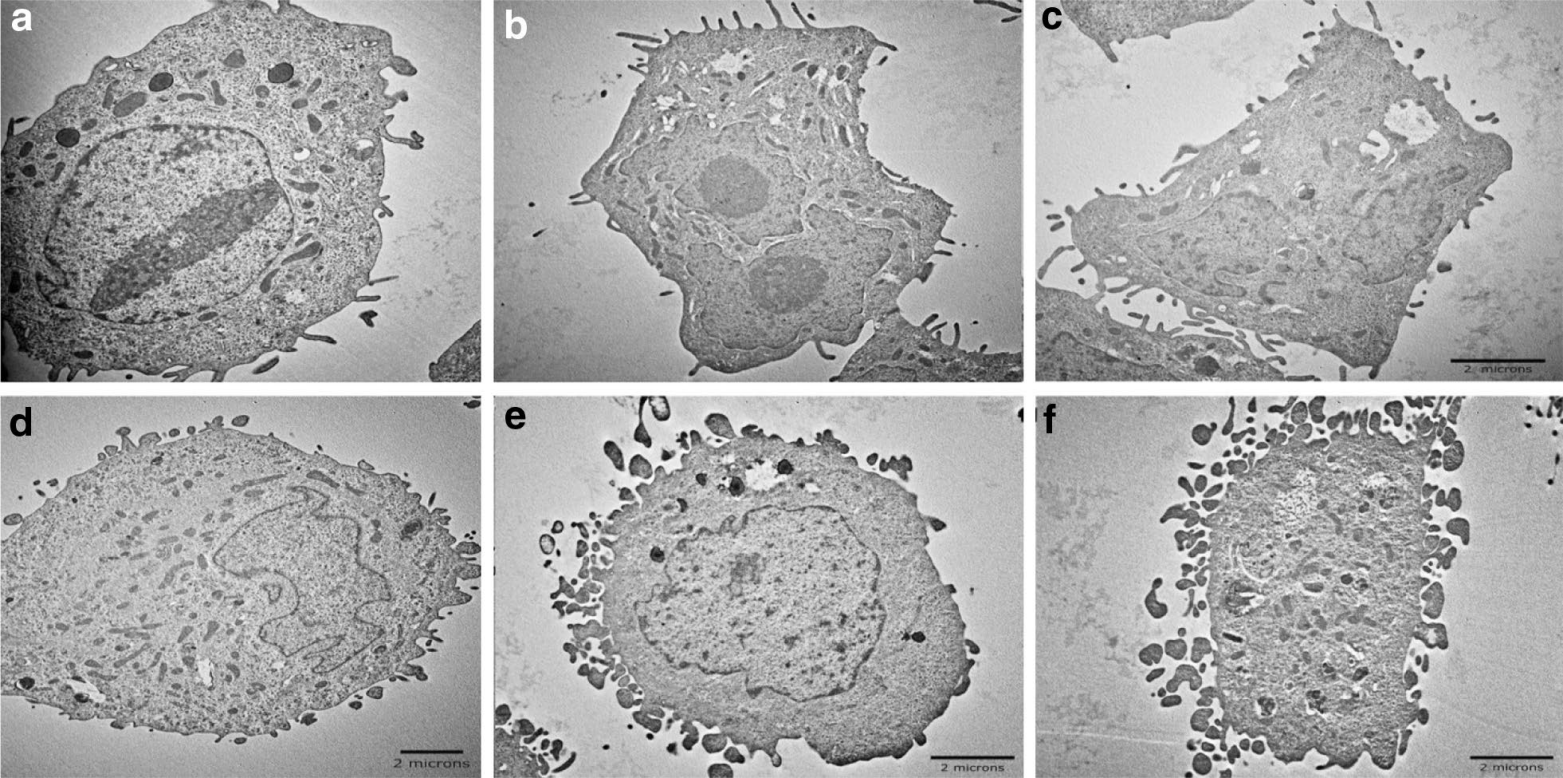
TEM images of H-184B5F5/M10 normal breast cells (**a**–**c**) and MDA-MB-231 breast cancer cells (**d**–**f**) cells, where **a** and **d** are control samples; **b** and **e** correspond to cells treated with US; **c** and **f** show the cells after combined treatment with US and magnetic NPs

To analyze the type of cell damage caused by US in combination with NPs, TEM images of H-184B5F5/M10 normal breast cells and MDA-MB-231 breast carcinoma cells were obtained (Fig. 4). Images a–c show the normal breast cells, and images d–f show the cancer cells. Images a and d are controls (no ultrasound exposure); images b and e are cells treated with ultrasound but not NPs. Images c and f depict cells treated with the combination of ultrasound and magnetic NPs. As shown in Fig. 4, the membranes of the cancer cells showed a more uneven structure after US treatment (Fig. 4e), particularly after treatment with US with NPs (Fig. 4f), than the membranes of the corresponding normal cells (Fig. 4b, c, respectively). No nanoparticles were found inside the cells.

### Analysis of nanoparticle uptake by the cells

To assess whether the enhanced cytotoxicity in lung and breast cancer cells is due to uptake of nanoparticles by cancer cells before US treatment, inductively coupled plasma mass spectrometry (ICP-MS) analysis of NP uptake by four cell lines (the breast cell lines MDA-MB-231 and MCF-10A and the lung cell lines A549 and BEAS-2B) was performed. The results showed similar uptake of both NPs by all cell types. However, the uptake of gold NPs was approximately 1 % of the amount initially added to cells by weight. There was a lack of cellular uptake of magnetic NPs by all cell lines. The conditions of cell sample preparation for the ICP-MS analysis were similar to the experimental conditions for the US treatment of cells.

Gold and super-paramagnetic iron oxide NPs possess a unique combination of physical and chemical properties, allowing these NPs to act as highly multifunctional anti-cancer agents. Gold NPs have been tested as drug carriers [8], photothermal contrast agents [5], and radio-sensitizers [9]. The location of gold NPs in the body can also be determined using photoacoustic imaging [10], or optical coherence tomography [11] in combination with X-ray imaging [12] or electron microscopy [13]. Magnetic iron oxide NPs have been used as magnetic resonance imaging (MRI) contrast agents [14], for magnetic hyperthermia treatment of cancer [15], and as magnetic drug carriers [16]. The advantages of biocompatible gold and magnetic NPs are their low toxicity and their ability to be used as contrast agents for various diagnostic methods for visualizing tumor tissues.

In addition to thermal effects [17], ultrasound radiation is known to produce mechanical pressure that results in acoustic streaming and cavitation within cellular systems [18, 19]. Free cavitation bubbles are formed in liquid media when the local liquid pressure drops below the saturated vapor pressure during the rarefaction period of the propagated ultrasound wave. The inertial cavitation process leads to the creation of shock waves, sonoluminescence and the formation of free radicals, which can irreversibly damage cells [20]. It is known that the threshold of inertial cavitation can be reduced by adding nucleation agents and changing the host fluid parameters [21, 22]. NPs may act as a permanent source of nucleation sites for cavitating bubbles, leading to the enhancement of inertial cavitation in culture media.

The extracellular effect of NPs was demonstrated by ICP-MS analyses. Both lung and breast cell lines exhibited low cellular uptake of gold NPs and a lack of cellular uptake for magnetic NPs. No visible magnetic nanoparticles were observed in breast cells based on TEM imaging. The fact that the most substantial cell damage was observed when NPs were added to the cell culture immediately before US treatment provides indirect evidence that the effects are due primarily to an extracellular effect. Increasing the cell incubation time with NPs resulted in higher viability of the cells after US exposure (data not shown).

It is known that malignant and normal cells differ in their metabolism and morphology [23]. The resistances of cancer and normal cells to physical stress are known to be different [24]. This conclusion is consistent with our experimental results and may explain why under the tension/compression force of ultrasound, cancer cells were preferentially damaged. This experiment shows that US exposure causes some cell damage to both normal and cancer cell types, but the effect is greater for cancer cells. This effect was demonstrated for A549 lung cancer cells in co-culture with their normal counterparts (BEAS-2B cells). The preferential destruction of cancer cell membranes was also observed in the TEM images of breast cells pre-incubated with magnetic NPs. However, the exact mechanism underlying the selective damage to cancer cells requires further investigation.

Although we used two pairs of “malignant/normal” lung and breast cell lines, two different types of NPs (gold and magnetic) and two types of cell treatment (in separate and co-cultured monolayers), selective killing of cancer cells was observed throughout the study. This finding may indicate that a combination of certain types of NPs with US fields could become a universal tool for cancer treatment.

Recently, we tried to apply NAUT to xenograft mouse model and observed the similar effect. Detailed will be published separately.

## Conclusions

In this study, we added NPs to cell cultures to determine whether the addition specifically enhanced the killing of cancer cells with low-intensity ultrasound. Our results showed that the addition of NPs indeed enhanced the killing of lung and breast cancer cells while largely sparing their normal counterparts in a co-culture system. Our discovery suggests that the power of US can be reduced relative to that used for HIFU ablation. For the treatment of malignant tumors in vivo, a decrease in US power would be valuable because it would allow for the selective destruction of cancer cells while sparing nearby healthy tissues and would reduce the reflection of US waves from bones and other reflective structures in the body.

## Methods

### Nanoparticle characterization

Gold nanoparticles (10 nm) were obtained from BBI International (Ted Pella, Redding, CA).

**Fig. 5:**
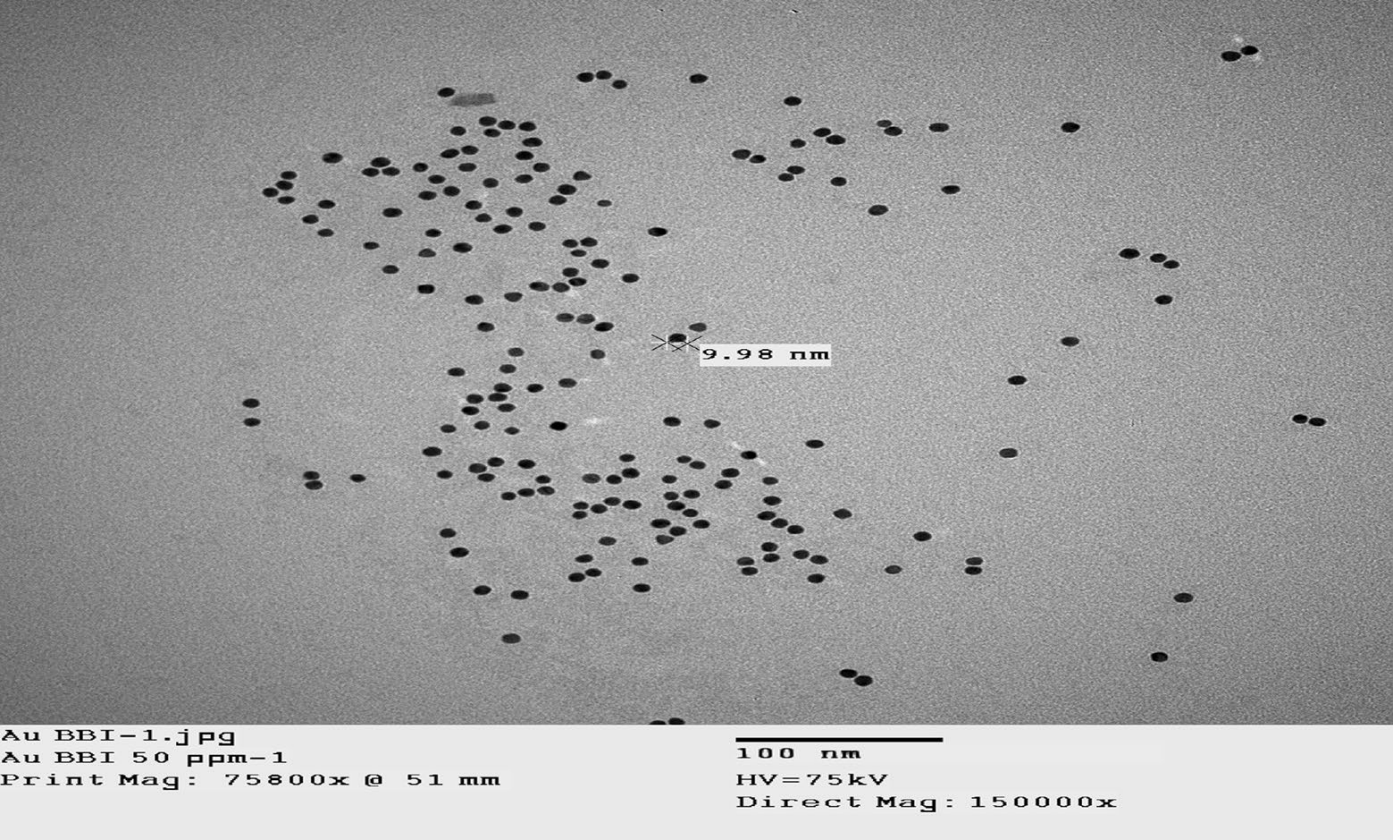
TEM image of nanogold used in this experiment

A TEM image of gold nanoparticles is shown in Fig. 5. Each individual nanoparticle can be clearly observed.

#### Preparation of magnetic nanoparticles

A mixed solution of ferrous and ferric ions in a molar ratio of 1:2 was prepared by dissolving 1.13 mmol FeCl_2_ × 4H_2_O and 2.26 mmol FeCl_3_ × 6H_2_O in an 80 cm^3^ aqueous solution of poly(vinylalcohol) (PVA) at 80 °C. PVA with an average molecular weight of 88,000 (Acros Organics) was used to obtain aqueous solutions at concentrations of up to 14 g dm^−3^. Magnetic iron oxide was precipitated by adding 0.5 mol dm^−3^ of NaOH to the above-mentioned mixture while heating to 80 °C and stirring with a magnetic stirrer. The mixture was subsequently incubated at 80 °C for 30 min and cooled to room temperature. The synthesis was performed in an oxygen-free environment.

#### Transmission electron microscopy

Transmission electron microscopy (TEM) was performed with a JEM-2100F (JEOL, Japan) instrument operating at an accelerating voltage of 75 kV. To prepare samples for TEM, a drop of the suspension (0.12 mg mL^−1^) was placed on a copper grid and dried under ambient conditions. The typical size of the metal core of magnetic NPs was approximately 4–6 nm and was narrowly distributed.

#### Dynamic light scattering

A DynaPro NanoStar light scattering spectrometer (Wyatt Technology, Taiwan) with an emission laser wavelength of 658 nm was used to measure the hydrodynamic diameter of the ferrofluid. All of the measurements were performed at an angle of 90°. The hydrodynamic size of magnetic NPs was estimated to be 140 nm.

#### Magnetometry

A drop of magnetic NPs (80 μL) was deposited onto a small piece of cotton and dried at room temperature for 3 days. The sample was inserted into a gelatin capsule and fixed inside to avoid any shifts. The saturation magnetization of the sample was measured with a superconducting quantum interference device (SQUID) magnetometer (Quantum Design, MPMS5) at room temperature (300 K) with a magnetic field of 7 T. The saturation magnetization was estimated to be 54.87 emu g^−1^ of iron mass. The anhysteretic magnetization curve corresponded to the superparamagnetic properties of the NPs.

#### Cell culture

Human A549 lung alveolar adenocarcinoma and BEAS-2B immortalized human bronchial epithelial cells were obtained from ATCC^®^ (Manassas, VA, USA). The A549 cells were cultured in Dulbecco’s modified Eagle’s medium (DMEM) with 10 % fetal bovine serum (FBS). Defined Keratinocyte-SFM culture medium (KSFM; Gibco^®^ Cell Culture, USA) was used for the BEAS-2B cells. The cells were cultured separately in a monolayer fashion with concentrations of 5 × 10^5^ cells mL^−1^ per well and in a co-culture (1:1) with a total concentration of 4 × 10^5^ cells mL^−1^. The detailed steps of the protocol are as follows.

Step 1 A549 and BEAS-2B were infected with lentivirus contain green and red fluorescent protein genes, respectively, and selected with neomycin (G418) at 800 μg per mL until fluorescent cells survived and grew to confluency. Green fluorescent protein (GFP) gene cDNA was constructed into a lentiviral packaging vector to become pLAS2w.GFP-C.Pneo, and red fluorescent protein (RFP) gene cDNA was constructed to become pLAS2w.RFP-C.Pneo.
Step 2 Each cell line was plated with 2.5 × 10^5^ cells in the same well of a 12-well plate with 10 % FBS DMEM medium overnight at 37 °C in 5 % CO_2_. Every well contained approximately 5 × 10^5^ cells before ultrasound treatment.
Step 3 Cells were washed twice with 1 mL PBS, and 1 mL 10 % FBS DMEM medium was added to ensure the volume before ultrasound treatment in each well in a 12-well plate. Fifty microliters of gold NP solution (BBI International Co.; 10 nm; Au stock concentration of 50 μg/mL) was added into the wells to achieve a final concentration of 2.5 μg/mL of Au NPs. In the other testing group, 50 μL of polyvinyl alcohol (PVA)-coated magnetic NPs (with a size of less than 7 nm) were prepared by chemical co-precipitation of Fe(II)/Fe(III) salts to reach a final iron concentration of 60 μg/mL in the cell suspension.
Step 4 *Ultrasound treatment* The Sonablate-500 (Focus Surgery Inc., USA) was chosen as the ultrasound source for cell irradiation. The dual-element self-focusing transducer was used in therapy mode with a 4-MHz resonant frequency and a 4-cm focal length. The probe was placed in a water tank with 4.5 L of degassed water for cell irradiation. Distilled water was obtained from a Millipore Q Synthesis A10 water purification system (resistivity = 18 MOhm cm^−1^, TOC = 3 ppb) and was degassed for 3 h using an on-line membrane vacuum degasser (ERC 3000 W/N, Endeavor Responsibility Challenge Co, Japan). The oxygen concentration in the water was measured prior to the experiments using an oxygen (dissolved) CHEMets Kit (K-7512, CHEMetrics Inc., USA) and was estimated to be 2–3 ppm. The water temperature in the tank was maintained in the range of 24–25 °C. The ultrasound power was adjusted using the software for the Sonablate-500. The shape of the ultrasound focal spot was a 3-mm-wide by 12-mm-high prolate spheroid. The transducer was operated in the scanning mode and irradiated 25 spots (5 × 5) in the 15 × 15-mm area under a well for 3 min 45 s. Thus, the treated region had a 3D 15 × 15 × 12-mm rectangular shape and was centered under the well. However, the center of the focal spot (with the maximum ultrasound intensity) was fixed at a distance of 3 mm under the culture plate’s surface. Each point of the plate surface was irradiated with US for 3 s. The size and location of the treated zone was similar for each well in the culture plate. The temperature of the culture medium in a well was measured after US treatment using a thermocouple, and the temperature change was found to be less than 0.1 °C. Thus, the average thermal effect during US treatment of cells was negligible. For US experiments, a power of 8 W was used, according to the read-out from the Sonablate-500 software. For the clinical treatment of prostate cancer, an US power of ~40 W is typically used. A corresponding total of 5.8 W radiated acoustic power was measured for an 8-W reading from the Sonablate software with a radiation force balance unit (UPM-DT-100AV, Ohmic Instruments Co.). A calibrated needle hydrophone (HNA-0400, Onda, CA, USA) was used to estimate the spatial-average temporal-average intensity, I_SATA_.

### Co-culture and cell analysis

For US-treated co-cultures of BEAS-2B and A549 cells, the numbers of attached cells were analyzed by optical microscopy. The attached cells were washed with 1 mL of PBS, followed by washing with an additional 1 mL of PBS with 0.1 mL of 0.4 % trypan blue for 5 min. Phase-contrast images of the attached cell monolayers were obtained via optical microscopy (Olympus IX71, USA) at 200× magnification and a digital camera (Olympus DP70). A mercury lamp (U-LH100HG) was used to produce separate fluorescence images of the cells modified with green and red fluorescent proteins. Cells stained blue were counted as dead cells under high magnification. Transparent cells were counted as live cells. The percentage of dead cells was determined by counting all the dead cells divided by the number of cells counted in a high-power field. Five fields were counted, with the means and standard deviations shown relative to those of the controls.

### Flow cytometry analysis

To collect ultrasound-treated cells for flow cytometry analysis, the medium was removed and washed in 0.5 mL PBS; 0.5 mL trypsin was added to detach the cells. Cells were harvested with treated medium, separated by pipetting several times and premixed with 1 μg mL^−1^ of propidium iodide (Sigma Aldrich, USA) before flow cytometry analysis by a BD FACS Canto II system (BD Biosciences, USA) using a 488-nm laser for excitation and a PE channel for fluorescence detection. The numbers of live cells (Q_4_) were measured for control and ultrasound-treated cells using BD FACS Diva software version 6.0.

### Transmission electron microscopy (TEM) of cells

Transmission electron microscopy was used to obtain images of H-184B5F5/M10 healthy breast cells and MDA-MB-231 breast cancer cells using the following procedure. The controls and US-treated cells were collected and fixed in 2.5 % glutaraldehyde and 0.1 M cacodylate buffer for 2 h at 4 °C. The cells were washed twice for 15 min in the cacodylate buffer. A secondary fixation was performed in 1 % osmium tetroxide for 1 h at 4 °C, followed by two additional 15-min washes in the same buffer. After dehydration, the material was embedded in Spurr’s resin. The resin was first diluted in acetone (1:1) and incubated at 4 °C with agitation for 2 h and then diluted in acetone (1:3) and incubated at 4 °C with agitation for 24 h. The pellet was transferred to pure Spurr’s resin and incubated at 60 °C for 48 h until completely polymerized. Sections measuring 70–90 nm were obtained using a Leica EM UC 7 ultra-microtome (Leica Microsystems GmbH). The sections were then placed on copper grids and were studied under a TEM (TEM Hitachi H-7000, High-Technologies Co. Japan).

### ICP-MS analysis of NP uptake by cells

Four cell lines (MDA-MB-231, MCF-10A, A549, and BEAS-2B) were seeded in 12-well plates (5 × 10^5^ cells per well) in different culture media and maintained in a humidified incubator for 24 h at 37 °C and 5 % CO_2_. MDA-MB-231 breast cancer cells were cultured with Dulbecco’s modified Eagle’s medium (DMEM) supplemented with 10 % fetal calf serum (FCS). MCF-10A normal breast cells were grown in DMEM/Ham’s F-12 with 5 % FBS, 10 μg mL^−1^ insulin, 5 μg mL^−1^ hydrocortisone, 20 ng mL^−1^ EGF and 100 ng mL^−1^ cholera toxin. A549 lung cancer cells were grown in RPMI 1640 medium supplemented with 10 % FBS. BEAS-2B normal lung cells were cultured in BEGM medium. The culture medium was replaced with 1 mL of serum-free DMEM. Gold or magnetic NPs were added to the cell suspensions at the same concentrations used for the ultrasound experiment. The cell cultures were incubated with NPs for 30 min at 37 °C with 5 % CO_2_ and washed three times with phosphate-buffered saline (PBS); 1 mL of concentrated HCl was added to every well, and the mixture was heated to 70 °C for 10 min. Then, samples were dissolved 10 times in double-distilled water and analyzed using ICP-MS (Thermo Scientific XSERIES 2) to determine the amount of gold and iron in the cells.

## Abbreviations

NAUT: nanoparticle-assisted ultrasound therapy
HIFU: high-intensity focused ultrasound
NPs: nanoparticles
US: ultrasound
ICP-MS: inductively coupled plasma mass spectrometry
MRI: magnetic resonance imaging
PVA: polyvinyl alcohol
TEM: transmission electron microscopy
SQUID: superconducting quantum interference device magnetometer
